# Ultrastrong catalyst-free polycrystalline diamond

**DOI:** 10.1038/s41598-020-79167-4

**Published:** 2020-12-16

**Authors:** Qiang Li, Guodong Zhan, Dong Li, Duanwei He, Timothy Eric Moellendick, Chinthaka P. Gooneratne, Alawi G. Alalsayednassir

**Affiliations:** 1 Institute of Atomic and Molecular Physics, Sichuan University, Chengdu, 610065 China; 2 Key Laboratory of High Energy Density Physics and Technology of Ministry of Education, Sichuan University, Chengdu, 610065 China; 3 Drilling Technology Division, Exploration and Petroleum Engineering Center - Advanced Research Center (EXPEC ARC), Saudi Aramco, Dhahran, 31311 Saudi Arabia; 4 Drilling Technical Department, Drilling and Workover, Saudi Aramco, Dhahran, 31311 Saudi Arabia

**Keywords:** Mechanical properties, Design, synthesis and processing

## Abstract

Diamond is the hardest naturally occurring material found on earth but single crystal diamond is brittle due to the nature of catastrophic cleavage fracture. Polycrystalline diamond compact (PDC) materials are made by high pressure and high temperature (HPHT) technology. PDC materials have been widely used in several industries. Wear resistance is a key material property that has long been pursued for its valuable industrial applications. However, the inevitable use of catalysts introduced by the conventional manufacturing process significantly reduces their end-use performance and limits many of their potential applications. In this work, an ultra-strong catalyst-free polycrystalline diamond compact material has been successfully synthesized through innovative ultra-high pressure and ultra-high temperature (UHPHT) technology. These results set up new industry records for wear resistance and thermal stability for PDC cutters utilized for drilling in the oil and gas industry. The new material also broke all single-crystal diamond indenters, suggesting that the new material is too hard to be measured by the current standard single-crystal diamond indentation method. This represents a major breakthrough in hard materials that can expand many potential scientific research and industrial applications.

## Introduction

Polycrystalline diamond compact (PDC) is widely used in nonferrous cutting materials, mining, and oil & gas drilling because of its outstanding properties such as high hardness, high thermal conductivity, high impact resistance, and wear resistance^1–5^. Traditionally, commercial PDCs are prepared by using a catalyst or binder metals^6,7^ or ceramics^8,9^ that effectively reduce the sintering conditions of diamond to approximately 5.5 GPa and 1400 °C. However, due to the use of binders, the hardness of the PDC is reduced to approximately 50–70 GPa^10–12^. Moreover, the metal catalyst binder (often referred to as cobalt catalyst) has an additional harmful effect when converting diamond into graphite at atmospheric pressure along with high temperatures generated from operations^13–15^. In addition, the cutting and drilling process exposes the PDC compact material to high stress and high temperatures environments, where PDC cutting edges with cobalt binders tend to produce microcracks and diamond particle falling/chipping when the stress is greater than the bonding strength of the diamond within the PDC compact, which greatly reduces the life of the PDC compact. The reason can be explained by the elastic modulus and thermal expansion coefficients between the cobalt metal binder and diamond not matching each other, which can lead to inconsistent volume changes between the diamond and binder in high stress and high temperature operating environments^16–20^. As a result, large stresses would be produced inside the PDC materials.

Hydrocarbon fuel energy has always been the main energy source in our society for decades. Global primary energy consumption is growing rapidly, mainly oil and gas. Polycrystalline diamond compact (PDC) drill bits are key drilling tools for oil exploration and drilling, with an annual market value of more than $4.5 billion for PDC cutters. Like semiconductor chips are key components for the manufacture of high performance computers, PDC cutters are key cutting or drilling components for the manufacture of PDC drill bits. The wear resistance of PDC cutters is the key performance indicator during drilling. Increasing wear resistance by 30% to 50% usually takes a decade. In our research, we developed a new strategy to manufacture new diamond cutting materials with ultra-high wear resistance, 300% more than the PDC cutters currently used in the industry, which will reduce technology investment by 50 years. However, drilling very hard, highly abrasive and interbedded formations is a very difficult challenge with today’s PDC bits^21,22^. Current PDC cutter technology do not provide sufficient wear or impact resistance, nor adequate thermal stability to survive this drilling environment to drill entire intervals in a single run, where use of multiple bits to drill a single hole section is not optimal for well economics, resulting in low ROP and short bit life^23–25^. The weakness in the current technology is due to the unavoidable use of cobalt catalyst to bind the diamonds that compose the PDC cutting structure^13,26^. Development of an ultra-strong and catalyst-free PDC cutter would be an ideal and game-changing drill bit technology, which provides a much needed technical solution and delivers a significant increase in performance, durability, and better well economics.

Ultra-high pressure and ultra-high temperature (UHPHT) large volume technology is cutting-edge for advanced superhard materials research. At present, the technology is mainly focused on the study of nanocrystalline diamonds; however, their industrial applications are limited by the low fracture toughness, size of tiny samples and/or high cost. In this study, we apply new UHPHT technologies to create centimeter-sized samples that are sufficient for industrial and scientific applications. Expanding the sample cavity is an important goal in the development of UHPHT devices, and its records are constantly refreshed. Irifune et al.^5^ made use of the Kawai-type large cavity static pressure device to successfully synthesize millimeter-grade nanocrystalline PDC under a high-pressure condition of about 15 GPa. Since then, after nearly 10 years of improvement, the size of synthetic nano-PDC has increased to the centimeter level^27–30^. Large tonnage high-pressure devices are required to obtain larger sample sizes and to ensure reasonable high-pressure efficiency. The high-pressure occurrence efficiency is mainly affected by the load loss in the transmission process, whether it is the mechanical structure of the assembly or the strength of the final stage of anvil material. The conveyor process consists of two aspects: (1) the combination of multistage loading, and (2) the final pressure chamber assembly. To improve pressure efficiency, an efficient multistage loading combination and suitable end-level pressure chamber components are required. For the first time, in the combination of multistage loading, we have developed a two-stage loading device^4^. This is directly integrated into the first six-sided cubic pressure chamber, eliminating the intermediate conversion process loaded by a single axis. Loading into three axes significantly improves the transmission efficiency of the load, compared to the 2–6–8 type loading based on uniaxial press technology.

In this report, we document the successful synthesis of catalyst-free PDC materials in centimeter size under an ultra-high pressure of 16 GPa and ultra-high temperature of 2300 °C using micron diamond powder as the starting material based on a hinge six-sided cubic (triaxial) press, where a centimeter cavity of single cylinder loading capacity is about 25 MN (2500 tons). A large sample dimension of over 10 mm in diameter and up to 6 mm in thickness has been achieved, which are sufficiently large enough and cost-effective to make parts and components for not only hydrocarbon drilling, but also a wide range of other industrial applications. In this paper, the physical and mechanical properties of new synthesized catalyst free polycrystalline diamond compact (CFPDC) materials, including wear resistance, hardness and thermal stability are systematically evaluated. By comparing the wear behavior and the evolution of cutting-edge microstructures to commercial PDC counterparts under the industry standard granite turning test method, the ultra-high wear resistance mechanism of CFPDC material is analyzed and discussed. The hardness of the new material was evaluated with the highest load forces, but it was found that the material broke all the single crystal diamond indenters, indicating that CFPDC is currently the hardest material in the world. Thermal stability was also evaluated to study its antioxidant properties under the in situ XRD machine at different air temperatures.

## Experimental

The starting diamond powder with grain sizes of 8 ~ 12 µm (M8/12, 99.9%, Zhengzhou Zhongnanjiete Superhard Materials Co., Ltd., China) was sintered at a pressure of 16 GPa and temperature of 2300 °C in a two-stage multi-anvil large volume high-pressure apparatus based on a DS6X25 MN cubic press^4^. The powders were treated in the vacuum furnace with a corundum container. When the vacuum chamber was pumped to 2 × 10^–4^ Torr, the sample was heated to 1200 °C at a rate of 15 °C min^−1^. After 90 min of maintaining the peak treating temperature, the sample was cooled to room temperature at 5 °C min^−1^. Next, the ends-treated powder was quickly packed into the cylindrical capsules made of metal tantalum foil (99.95%, Alfa Aesar, Ward Hill, MA) with a diameter of 13 mm and thickness of 6.3 mm, which was then put in the high-pressure apparatus. Magnesium oxide (MgO, 99.99% purity) doped with 5% Cr_2_O_3_ was used as a pressure media. High pressure assembles 36/20 (octahedron edge length/truncated edge length, designed by the Laboratory of High Pressure Science and Technology, Sichuan University) were used for generating pressures. The sample packaged with tantalum foil was placed in a MgO sleeve. The tantalum (Ta) tube was used as the heater, and zirconium dioxide (ZrO_2_) sleeve as the thermal insulator. The high-pressure cell temperature was measured by W97Re3–W75Re25 thermocouples and pressure was calibrated by the pressure-induced phase transitions of Bi, ZnTe, and ZnS. Samples were compressed to the desired values before heating and then treated from 1000 to 2300 °C under a pressure of 16 GPa for 10 min. Finally, the samples were cooled to room temperature at a rate of 50 °C min^−1^. Then, the pressure was released in 120 min. The capsules extracted from the multi-anvil assembles were opened and treated with acid to remove the tantalum foil. After that, the samples were washed in water and then in ethanol using an ultrasonic bath for characterization.

An x-ray diffractometer (XRD; λ = 0.15406 nm, Fangyuan DX-2500, China) was applied to determine the phase and structure of the starting diamond powder, CFPDC and commercial PDC materials. Raman scattering spectra of the starting diamond powder, sintered polycrystalline diamonds and commercial PDC materials were obtained at room temperature and pressure in a confocal Raman system in which a solid-state laser (532 nm, RGB laser system, NovaPro 300 mW, Germany) and a triple grating monochromator (Andor Shamrock SR-303i-B, EU) with an attached EMCCD (ANDOR Newton DU970P-UVB, EU) were used. The average grain size of samples and starting powder was investigated using the backscattered electron image analysis (BSE; JSM-IT500HR, Japan) and scanning electron microscope imaging (SEM; JSM-IT500HR, Japan). A numerical control lathe (Model SK50P/750, Baoji, China) was used to carry out the wear resistance performance tests. The hardness of polished specimens was tested with different loading forces up to 9.8 N and a fixed indentation time of 15 s by a Vickers hardness tester (FV-700, Japanese future technology). The thermal stability and oxidation resistance were evaluated under a high-temperature in-situ XRD machine (XRD; λ = 0.15406 nm, Fangyuan DX-2700, China) at different temperatures, from room temperature to 1400 °C.

## Results and discussion

**Figure 1:**
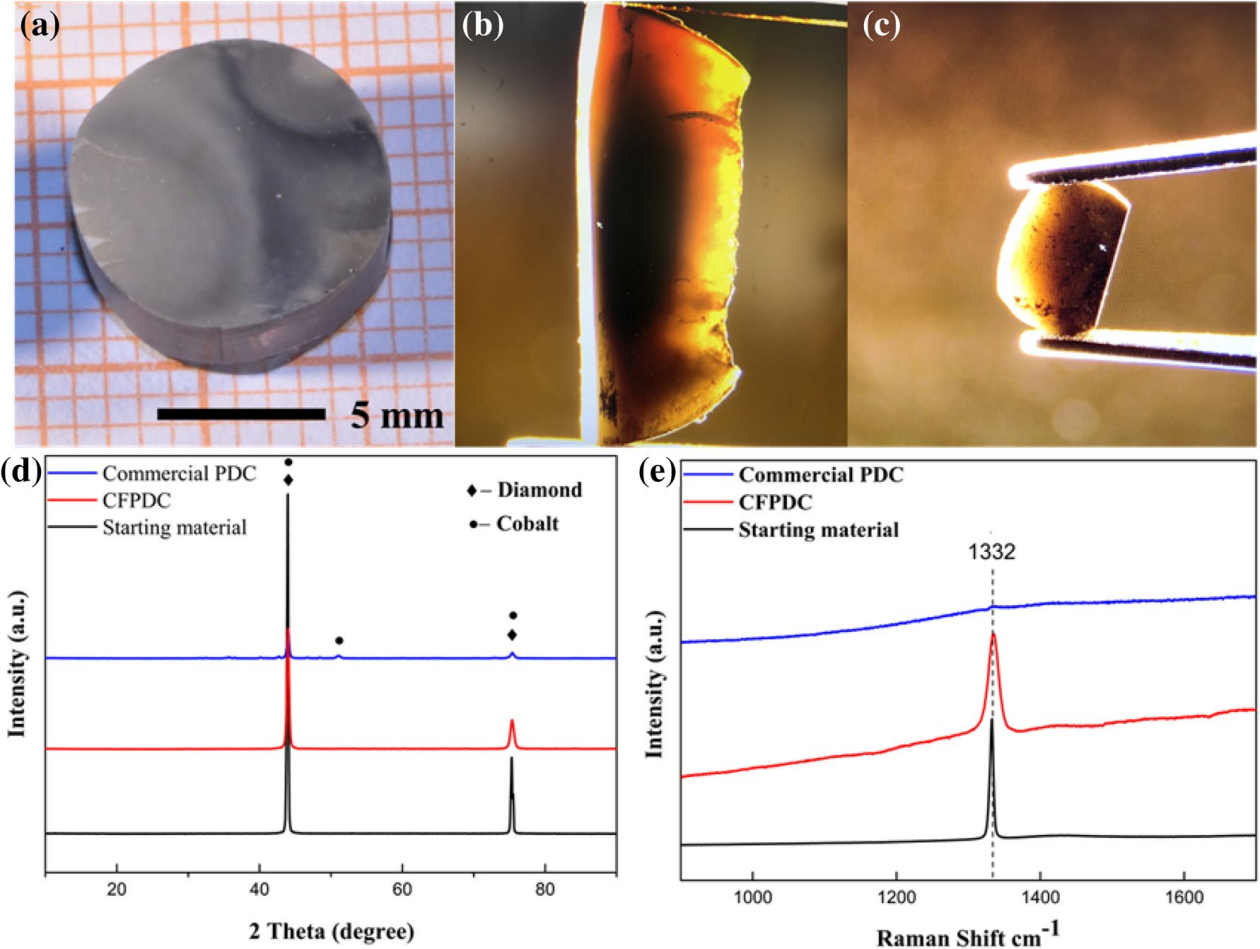
Catalyst-free polycrystalline diamond compact. (**a**) Optical picture of the polished CFPDC sample prepared under 16 GPa and 2300 °C (11 mm in diameter and 6 mm thick). (**b**,**c**) Optical pictures taken when a visible light beam passes the CFPDC bulk sample. (**d**) XRD patterns of starting powder, sintered CFPDC and commercial PDC samples. (**e**) Raman spectra patterns of starting powder, sintered CFPDC and commercial PDC samples. The analytical results show that the starting material and sintered CFPDC samples are pure and well-crystallized diamonds. The commercial PDC sample contains not only a diamond phase but also a cobalt binder. The measured commercial PDC peak is relatively weak due to the absorption of Raman light by cobalt.

Microstructural analysis can be very intuitive to observe the microscopic morphology of new material samples. To detect and compare the microstructure of the CFPDC and commercial PDC samples, the detailed microstructure of the synthesized and polished samples was investigated by Scanning Electron Microscopy (SEM). SEM and backscattered electron image (BSE) analyses showed that the diamond grain size is approximately 10 µm for both the starting diamond powder and the CFPDC material. Commercial PDC materials with the same grain size were also selected for comparison. Figure 1a shows the polished CFPDC sample prepared under 16 GPa and 2300 °C, which has a dimension of 11 mm in diameter and 6 mm in thickness. These dimensions fully meet the requirements for making industrial turning and drilling tools. It is worth noting that the samples are in a translucent state as shown in Fig. 1b and c, which are optical pictures taken when a visible light beam travelled past the bulk CFPDC sample. Figure 1d and e show the XRD patterns and Raman spectroscopic patterns of the starting powder, CFPDC and commercial PDC. It can be seen that both the starting material and sintered CFPDC materials are pure and well-crystallized diamonds. The commercial PDC materials used for the log turning test contains not only a diamond phase but also a cobalt binder.

**Figure 2:**
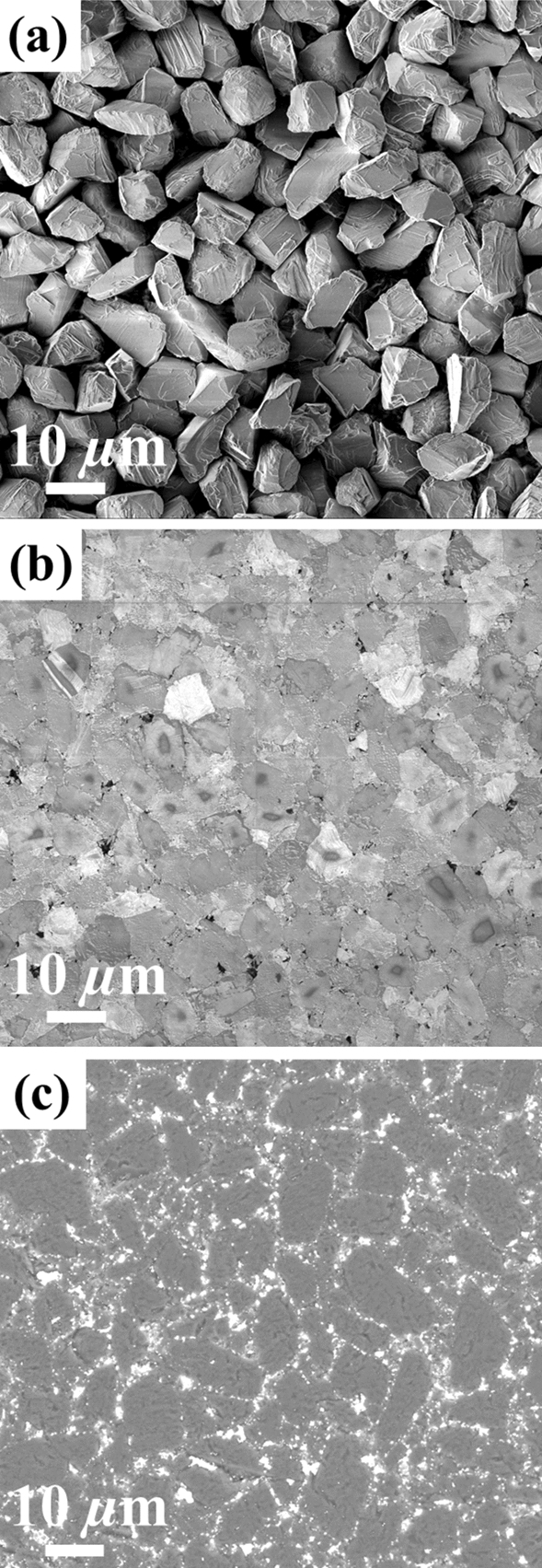
Microstructures of starting diamond powder, polished CFPDC, and polished commercial PDC samples. (**a**) SEM image of starting diamond powder; (**b**) SEM image of the CFPDC sample; (**c**) BSE analysis of the commercial PDC sample. The dark gray region is diamond and the bright region is residual cobalt catalyst. These images show that the diamond grain size is all about 10 µm for both the starting diamond powder and the CFPDC samples, and the commercial PDC sample with the same grain size are also selected for comparison.

In order to effectively avoid the effect of grain size on the turning log results, all the initial diamond powder, CFPDC and commercial PDC compacts have an average grain size of about 10 µm as shown in Fig. 2a–c. The dark gray region in Fig. 2c is diamond and the bright region is residual cobalt catalyst from the BSE analysis of the commercial PDC. Therefore, the residual cobalt catalyst is dispersed at the diamond grain boundary in the commercial PDC, which can break the diamond to diamond bonding during harsh cutting or drilling in high temperature and high stress environments.

**Figure 3:**
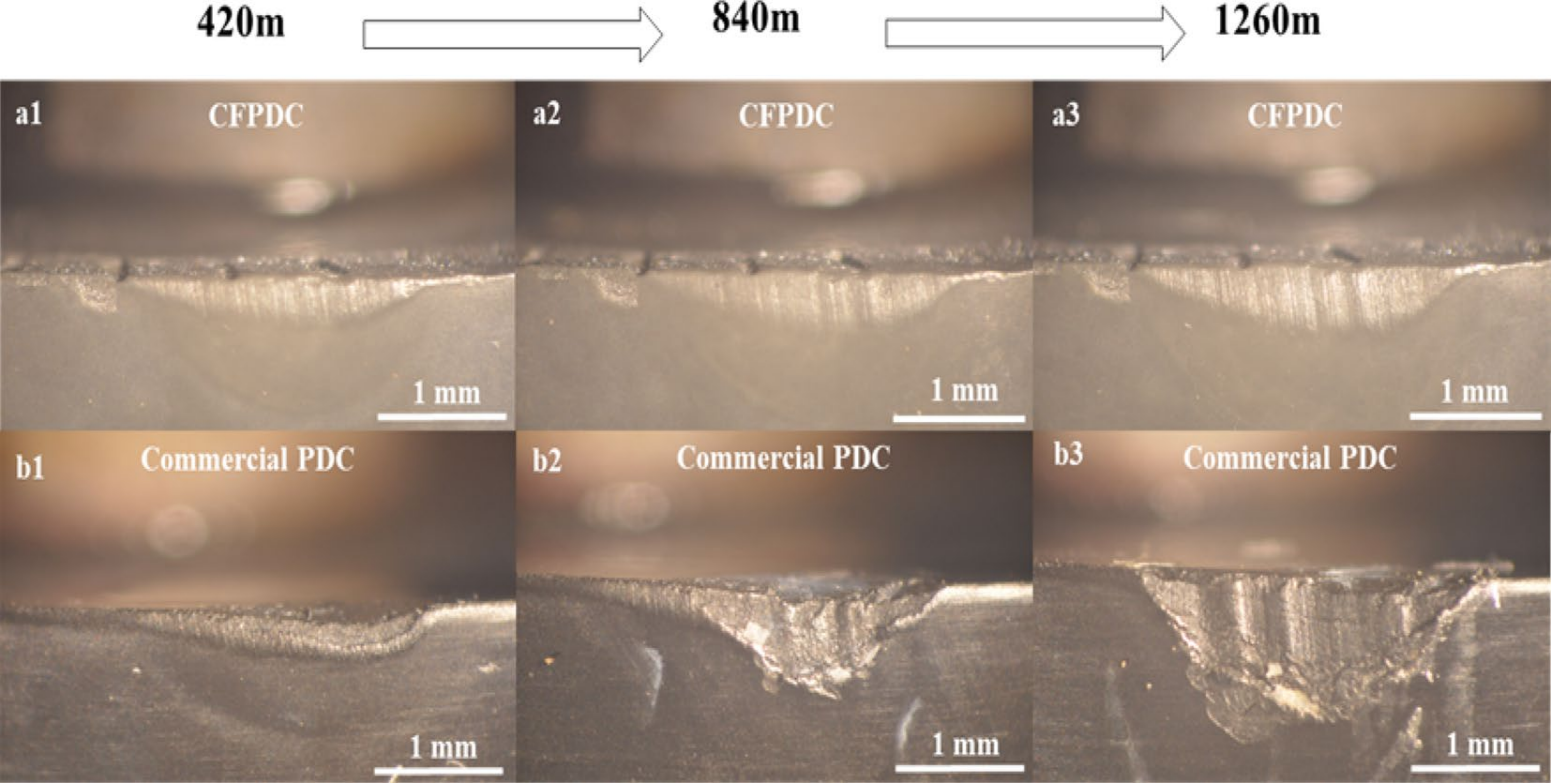
Wear flats of CFPDC and Commercial PDC samples. Optical pictures for the cutting edge of CFPDC sample (upper) with turning length of (**a1**) 420 m, (**a2**) 840 m, and (**a3**) 1260 m; and pictures for the cutting edge of commercial PDC sample (lower) with a cutting length of (**b1**) 420 m, (**b2**) 840 m, and (**b3**) 1260 m, respectively.

The cutting performance of samples was evaluated in a numerical control lathe by turning a granite log, which is the most widely distributed material on the earth's crust with a high hardness, high wear resistance, and low thermal conductivity. The cutting parameters utilized for the granite log turning test were as follows: cutting speed (Vc) of 100 m/min, depth of cut (Ap) of 0.5 mm, and feed rate (f) of 0.4 mm/rev. The CFPDC and the commercial PDC samples were processed into cylindrical cutting tools with a diameter of 11 mm and height of 6 mm. Figure 3 shows the optical images of the cutting edge of CFPDC sample and commercial PDC when the cutting length is 420 m, 840 m, and 1260 m, respectively. We can clearly see that the cutting-edge wear areas of commercial PDC sample are uneven and significantly larger than those for CFPDC sample, especially when the turning length reached 840 m and 1260 m. There is even a piece of commercial PDC sample falling off the cutting edge as shown in Fig. 3b3. On the contrary, the wearing surface of cutting edge for the CFPDC sample is relatively smooth as shown in the Fig. 3a1–a3.

**Figure 4:**
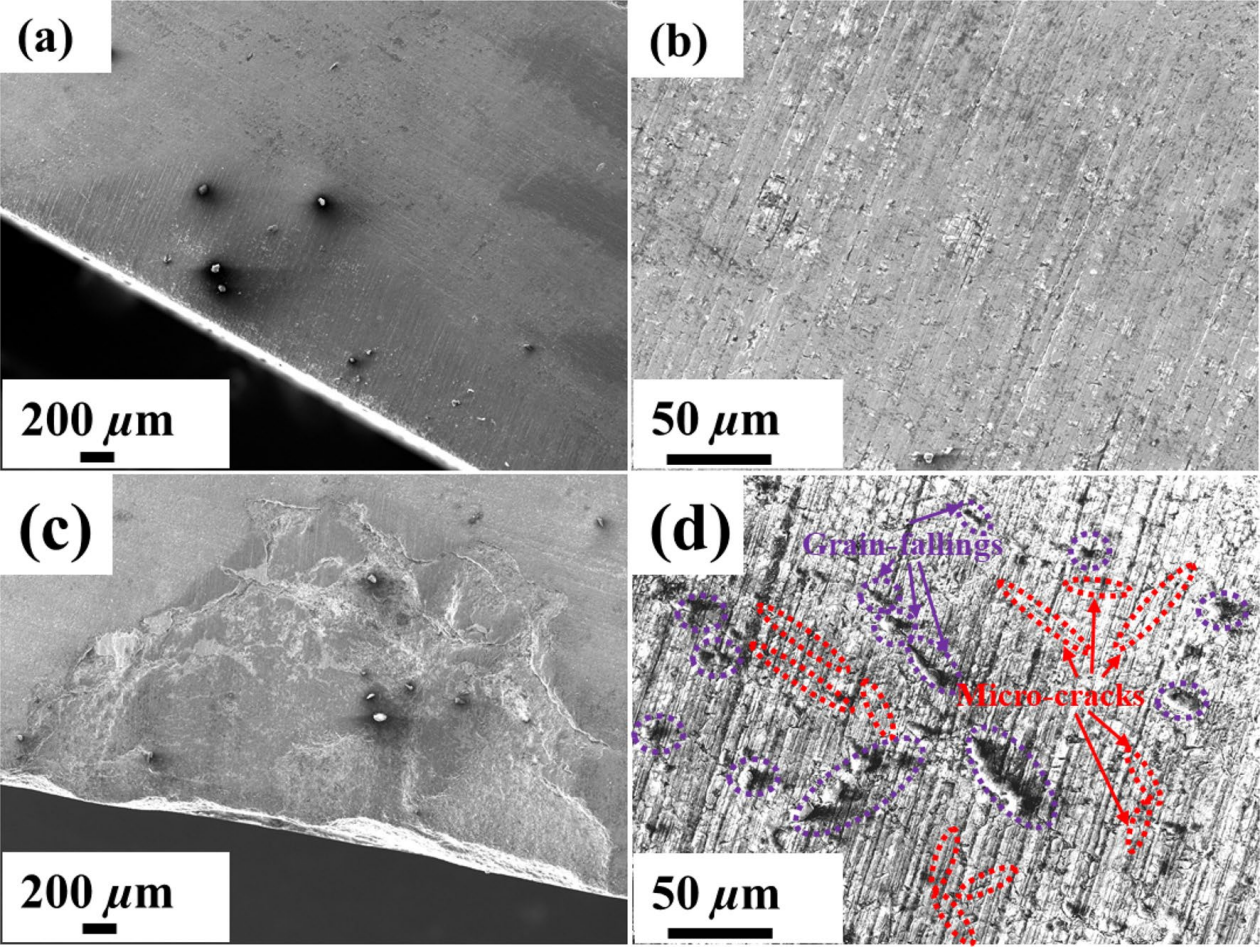
Morphology of Wear flat Surfaces. SEM image of the wear surface for the cutting edges of CFPDC sample (**a**,**b**) and commercial PDC sample (**c**,**d**) with a cutting length of 1260 m. Micro-cracks are marked with red circles while grain-fallings are marked with purple circles in (**d**).

**Figure 5:**
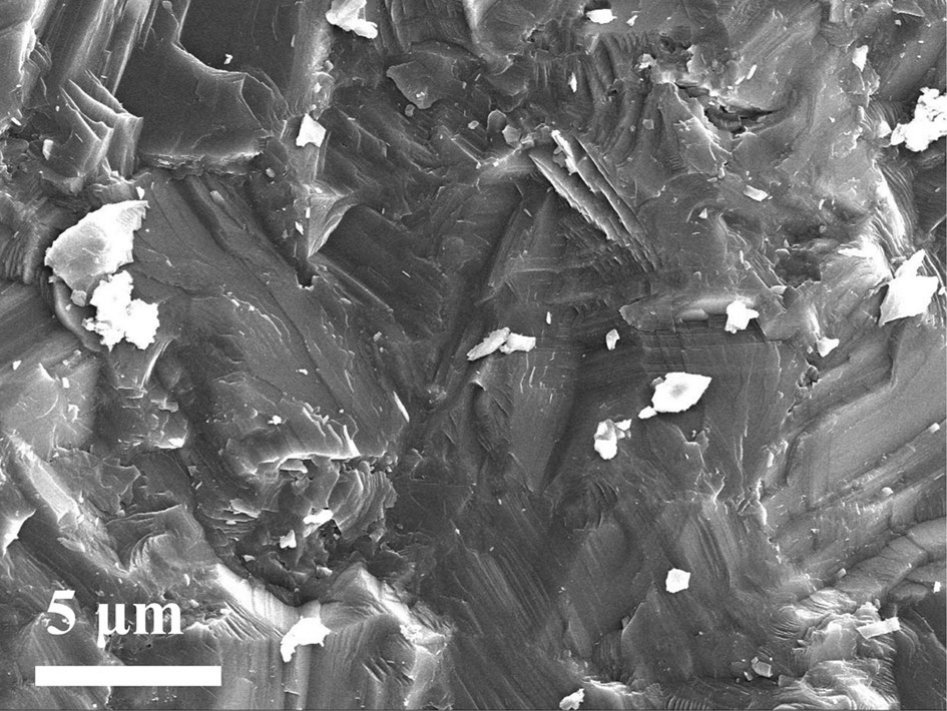
SEM Fracture Surface. SEM image of the fracture surface of the CFPDC material showing transgranular fracture failure mode, clearly demonstrating extreme strong diamond to diamond bonding.

After a turning length of 1,260 m, the wear surface of cutting edges for our prepared CFPDC and commercial PDC samples were further observed using SEM and BSE as shown Fig. 4a–c. The commercial PDC sample suffered serious breakage and chipping damage after 1260 m of cutting, and there are also many micro-cracks and grain-falling in the cutting edge of the sample as shown in Fig. 4c and d. This is because of the large mismatch of the elastic modulus and thermal expansion coefficient between the diamond and cobalt catalyst, and the graphitization of diamond by catalytic reaction of cobalt catalyst, owing to the increasing temperature and high stress at the cutting point. On the contrary, the CFPDC sample, which only has few observed defects on the cutting edge, demonstrated outstanding wear resistance as shown in Fig. 4a and b, since the CFPDC sample has no metal catalyst and possesses strong diamond-diamond bonding among grains. This is further demonstrated by the transgranular fracture failure mode on the fracture surface, as shown in Fig. 5.

The wear resistance of the CFPDC and commercial PDC samples is evaluated using the abrasion wear ratio, *E*, and the wear rate or ratio is calculated using the following equation:

1 $$E=\frac{V1}{V2}$$

where *E* is abrasion ratio; *V*_*1*_ is the volume of worn granite, (mm^3^); *V*_*2*_ is the volume of worn cutting tools (mm^3^).

**Figure 6:**
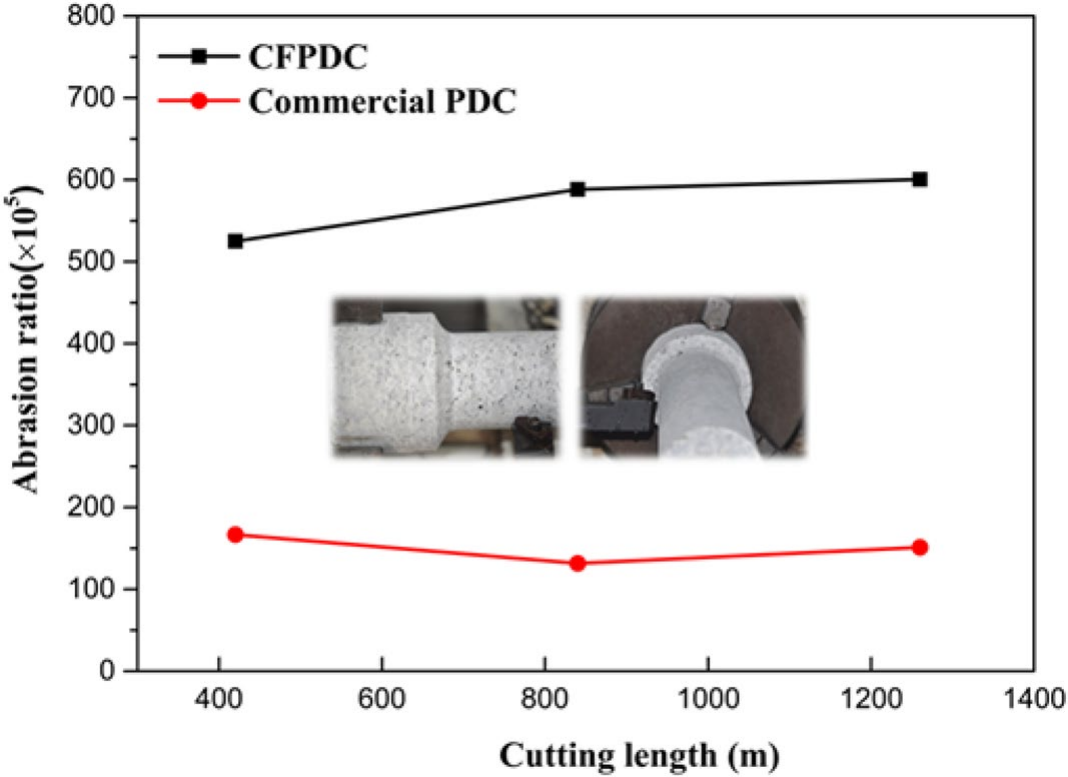
Wear ratios with cutting length. Abrasion ratio at different cutting lengths for commercial PDC and CFPDC samples, and inset is the optical picture to show the turning experiment set up.

Figure 6 shows the results of the wear ratio of CFPDC and commercial PDC samples for different cutting lengths. The wear ratio of CFPDC sample is observed to be at a stable level with the increase of cutting length as there is no falling off of diamond blocks in the process of turning granite testing. The wear rate of the commercial PDC is the opposite, as the heat expansion coefficient and elastic modulus mismatch between diamond and cobalt cause severe chip damage. In addition, the turning test results show that the average wear of the CFPDC sample is more than three times higher than that of the commercial PDC sample. As a result, the CFPDC sample has exceptional wear resistance compared to the best diamond materials currently used in the industry. The extraordinary wear resistance of the CFPDC material is directly related to its ultrahigh hardness. It was found that the length of the Vickers indentation on the compact surface of the polished CFPDC was too small to be measured, even if we increased the load force to 9.8 N with a dwelling time of 15 s, suggesting that the hardness value of the new material would be hundreds of GPa, or infinite if we would apply the Vickers’s hardness equation with near zero indentation or impression area. This is significantly different from our previous work on 14-GPa PDC materials^4^. In the previous paper, synthesis treatments are applied at constant pressures of 14 GPa in different synthesis temperatures ranging from 1300 to 2000 °C. The best or optimal performance was found at 1900 °C. When the temperature reached and exceeded 2000 °C, its hardness decreased, even though a temperature difference was only 100 °C, indicating that temperature plays an important role in material performance, especially under extreme high pressure conditions. In this study, we utilized synthesis or processing temperatures of up to 2300 °C, 400 °C higher than the 14-GPa/1900 °C material, which under previous conditions would melt most of the heater material. Such an extreme high temperature processing pose a significant challenge to high pressure cell or chamber design and uniform temperature control, as well expose the limitations of the pressure chamber or cell materials. Therefore, in this paper, we addressed these challenges with new materials and cell designs, maintaining a constant synthetic pressure of 16 GPa under an extreme high temperature of 2300 °C to obtain completely dense and ultrastrong materials.

**Figure 7:**
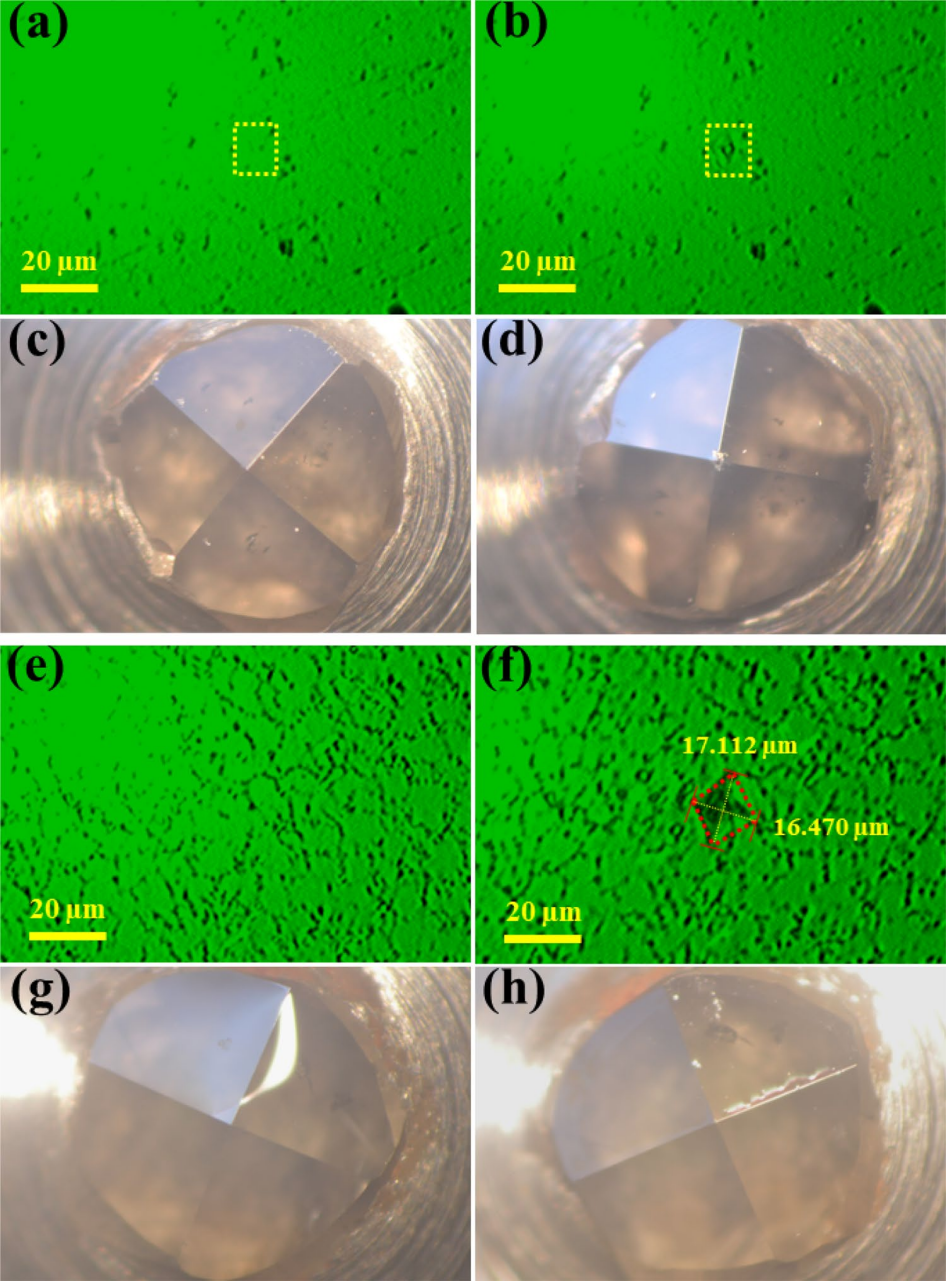
Hardness. Optical picture of the polished CFPDC and commercial PDC sample surfaces before (**a**,**e**) and after (**b**,**f**) hardness test with a load of 9.8 N and dwelling time of 15 s, respectively. Photographs of Vickers indenter tip before (**c**,**g**) and after (**d**,**h**) hardness test of CFPDC and commercial PDC samples under a load of 9.8 N and dwelling time of 15 s, respectively. We could draw a conclusion that the hardness of the CFPDC sample had exceeded the Vickers hardness limit of single crystal diamond, while the hardness of commercial PDC sample is only about 64 GPa.

Figure 7a, e and b, f show the surfaces Vickers hardness indentation before and after the polished CFPDC sample and commercial PDC sample were applied a highest load of 9.8 N and a dwelling time of 15 s, respectively. Figure 7c and d show the Vickers hardness (*Hv*) indentions before testing with a perfect standard square-pyramidal diamond indenter and a broken indenter after indented into the CFPDC sample, respectively. On the other hand, the standard square-pyramidal diamond indenter is perfect before (Fig. 7g) and even after (Fig. 7h) the hardness test of a commercial PDC sample. The results showed that the hardness of the CFPDC sample had exceeded the Vickers hardness limit of single crystal embedded diamond (120 GPa)^31^, indicating one of the world’s hardest materials to date, whereas the hardness of commercial PDC material is only about 64 GPa due to the residual cobalt binder found in commercial PDC materials. The observed superior hardness of CFPDC samples can be attributed to nanostructure defects induced by high-pressure working hardening, such as stacked nanolamellaes, stacked faults, and twin microstructures^4^.

**Figure 8:**
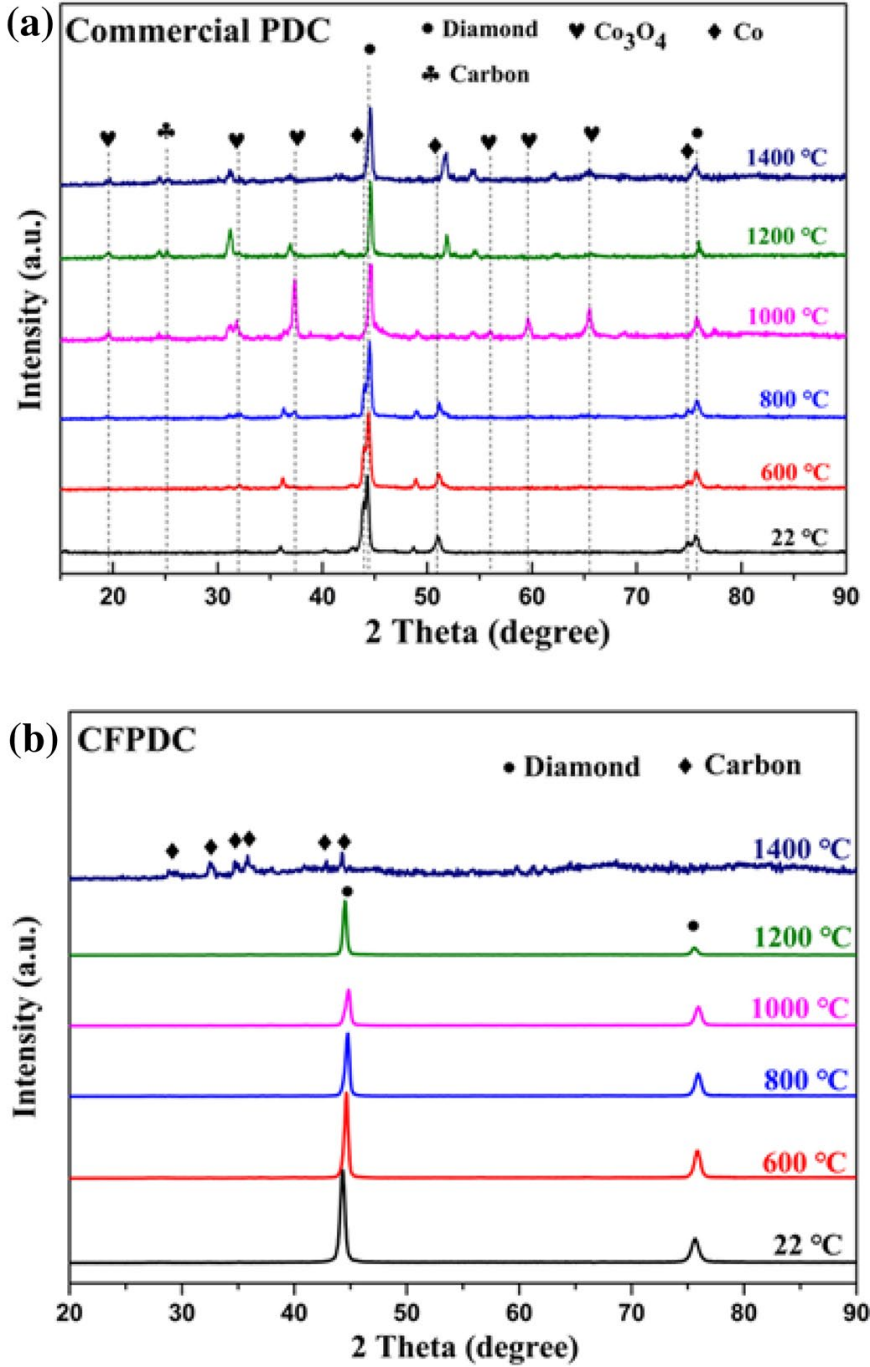
Thermal Stability and Oxidation Resistance. In-situ high temperature XRD patterns of commercial PDC and CFPDC samples at different temperatures ranging from 22 to 1400 °C in air, (**a**) Commercial PDC sample, (**b**) CFPDC sample The results of In-situ high temperature XRD indicate that the commercial PDC sample begins to oxidize at 600 °C, while CFPDC sample can be stable up to 1200 °C.

High temperature thermal stability and oxidation resistance are important to the materials end-use applications, especially in hard and abrasive formation drilling in the petroleum industry. The thermal stability and oxidation resistance tests were carried out under in-situ XRD at different temperatures from room temperature to 1400 °C. For comparison, a commercial PDC material was also tested. As seen from Fig. 8a, the starting oxidation temperature of commercial PDC is about 600 °C. A severe degradation can occur at temperatures of over 800 ℃. On the other hand, the new CFPDC sample, was found to be stable up to 1200 °C (Fig. 8b)—the highest recorded in the industry, which is much higher than that of natural diamond (~ 800 °C)^32,33^, nano-grained diamond (~ 680 °C)^34^, nanotwinned diamond (~ 1056 °C)^35^ and commercial PDC (~ 600 °C)^5,36^.

## Conclusion

Catalyst-free polycrystalline diamond compact (CFPDC) materials with ultra-high wear resistance have been successfully synthesized under an extreme high pressure of 16 GPa and ultra-high temperature of 2300 °C. The wear resistance, hardness, and thermal stability have been symmetrically evaluated. The results show that the new material possesses an exceptional high wear resistance—more than 300% higher than that of the best commercial PDC materials used in the industry. The material also broke all indenters during the hardness testing when the loading force was larger than 9.8 N, indicating one of the world hardest materials to date. Furthermore, the material exhibits the highest thermal stability and oxidation resistance of up to a temperature of 1200 °C. The exceptional combination of the highest hardness, highest wear resistance, and highest thermal stability of the new CFPDC material is expected to play an important role in scientific research and in industries such as oil & gas exploration and drilling, tool cutting materials, semiconductor, aerospace explorations, and innovative 4IR manufacturing. The UHPHT processes between our CFPDC (i.e., micro-sized polycrystalline diamond—MPD) and NPD (nanocrystalline polycrystalline diamond) are two distinguished technologies or directions with totally different sintering mechanisms. Both are important to scientific research and industry applications.
